# Effect of High-Intensity Interval Training on Functional Movement in Older Adults: A Systematic Review and Meta-analysis

**DOI:** 10.1186/s40798-023-00551-1

**Published:** 2023-01-15

**Authors:** Guy Stern, Stelios G. Psycharakis, Shaun M. Phillips

**Affiliations:** 1 The University of Edinburgh, St Leonard’s Land, Holyrood Road, Edinburgh, EH8 8AQ UK; 2 Human Performance Science Research Group, Moray House School of Education & Sport, Institute for Sport, PE and Health Sciences, Edinburgh, Scotland; 3 Biomechanics, Moray House School of Education & Sport, Institute for Sport, PE and Health Sciences, Edinburgh, Scotland; 4 Sport & Exercise Physiology, Institute for Sport, PE and Health Sciences, Edinburgh, Scotland

**Keywords:** High-intensity interval training, Moderate-intensity continuous training, Functional movement, Older adults

## Abstract

**Background:**

Preserving physiological functional capacity (PFC), the ability to perform the activities of daily life, and the ease with which they can be performed, in older adults, defined for this study as ≥ 50 years of age, is an important consideration for maintaining health and independence through the ageing process. Physical activity, and exercise training in particular, has been positively associated with improvement in PFC. In addition to improving aerobic and anaerobic capacity, promoting and preserving functional movement as a component of PFC is an important goal of physical activity, especially for older adults. High-intensity interval training (HIIT), an exercise protocol where repeated bouts of increased intensity are interspersed with active or passive recovery periods, has often been studied as an alternative to traditional moderate-intensity continuous training (MICT) exercise, where a continuous intensity is maintained throughout the exercise session. A large body of research has determined that both types of exercise programme are effective in improving measures of aerobic and anaerobic fitness in older adults. However, the effect of the two exercise modalities on functional movement has most often been a secondary outcome, with a range of observational techniques applied for measurement.

**Objectives:**

The primary objective of this research is to systematically review and meta-analyse published studies of HIIT interventions that measured functional movement in older adults to conclude if HIIT is effective for improving functional movement. A secondary objective is to determine if there are significant differences between HIIT and MICT effect on functional movement.

**Methods:**

A search strategy of terms locating studies of HIIT interventions, functional movement outcome measures, and older adult population samples was executed on seven digital databases. Randomized and pair-matched trials of > 2 weeks were considered for inclusion. Studies of participants with neurological impairment or studies using combined exercise modality were rejected. Standardized mean difference for functional movement outcome measures was calculated. A meta-analysis of the included studies and subgroups was performed along with study quality (risk of bias and publication bias) evaluation.

**Results:**

A total of 18 studies were included in random effects model pooled analysis. Subgroup analysis of HIIT versus MICT on functional movement showed a trivial effect in favour of HIIT (ES 0.13, 95% CI [−0.06, 0.33] *p* = 0.18) and did not achieve statistical significance. However, HIIT showed a medium, statistically significant favourable effect on functional movement versus non-intervention control (ES = 0.60 95% CI [0.24, 0.95] *p* = 0.001). Further subgroups analysis using singular and multiple functional movement outcome measures showed similar results.

**Conclusion:**

This meta-analysis indicates that HIIT interventions in older adults may be effective at promoting improvements in functional movement, though it is unclear whether HIIT is superior to MICT.

## Key Points

- High-intensity interval training is an effective exercise regime for improving functional movement in older adults.
- The results of HIIT versus moderate-intensity continuous training on functional movement are inconclusive.
- Future studies should use more comprehensive measures to assess functional movement than those oriented to the frail and elderly.

## Introduction

### Functional Movement

The ability of the older adult population, usually classified in guidelines as ≥ 65 years of age [1], to perform the physical tasks of daily life depends on the preservation of functional movement [2]. Functional movement is defined as fundamental movement patterns that require a balance of mobility and stability (including neuromuscular/motor control) used in the performance of basic locomotor, manipulative, and stabilizing movements [3]. Functional movement facilitates physiological functional capacity (PFC), the ability to perform the activities of daily life, and the ease with which they can be performed [4, 5].

### Functional Movement Measurement

The definition of PFC provides an impractical basis for direct measurement, and the measurement of functional movement, particularly in older adults, has not been standardized in the same way as, for example the measurement of aerobic (V̇O_2max_) or anaerobic (peak power output, PPO) capacity. Two test batteries, or components thereof, have most often been used as indicative evaluations of functional movement in older adults. The short physical performance battery (SPPB) includes five physical tests that assess lower extremity function: three timed static standing tasks with feet in different positions and two timed mobility tasks: an eight-foot timed walk and a five-repetition sit-to-stand task [6]. Though originally designed as an indicator of mortality and independence, the SPPB has been validated as a measure of functional status in older adults. The senior fitness test [7] was developed as a functional fitness test battery to assess physiological parameters of mobility and independence in older adults. The test incorporates two static indicators of flexibility: a seated chair sit and reach and shoulder flexibility back scratch test, and four dynamic tasks: the 30-s chair sit-to-stand (STS), seated arm curl, six-minute walk test (6MWT), and eight-foot (timed) up and go (TUG). The tests have been subsequently used to develop performance standards for mobility and independence in older adults [8]. The dynamic components of these test batteries are more commonly used as they seem best suited to measure functional movement, though the selection is often arbitrary. Other functional movement evaluations exist, for example, the functional movement screening (FMS) [9] but are rarely applied to intervention studies in older adults.

### Change in Functional Movement With Age

Functional movement in older adults is typically evaluated by the above-referenced tests which largely concentrate on lower-body skeletal muscle in the sagittal plane rather than assessments of whole-body movements in multiple planes. Consequently, there is less information and some debate about the rate of change in functional movement through the ageing process. Using five-year stratified age groups, the normative values of FMS [9] generally indicate a rate of decline of about 7% per decade (*n* = 622) [10] from peak at about age 35–40 years [4] through age 64. In a much smaller sample (*n* = 108) Mitchell et al*.* [11] observed a 27.4% lower FMS score between 50- and 54-year-old and 70- and 74-year-old groups. Similarly, functional movement measured by the senior fitness test [7] has shown a 32.2% decline from 60 to 90 years, with the rate of change more than doubling in the eighth and ninth decades [8]. This implies a period of age 50–70 years where functional movement decline might be relatively linear, followed by an accelerated or even quadratic deterioration in later years.

### The Relationship Between Physical Activity and Functional Movement

Like other physical fitness parameters, functional movement can be mediated by structured exercise. Physical activity (PA) guides and references [1, 2] have recommended implementing the principles of overload, specificity, and functional relevance (muscle activation similar to everyday activities), to combat deterioration in the functional movement leading to impairment and functional limitations with advancing age. Farrell et al*.* [12] found significant (*p* < 0.0001) positive correlations between functional movement and level of PA (*r* = 0.252) and frequency of resistance training (*r* = 0.208) in adults aged ≥ 55 years, though the relatively low coefficients might indicate that it is still unclear what mode, intensity and duration of exercise are most effective for promoting functional movement in older adults.

### Physical Activity Recommendations for Older Adults

According to the current American College of Sports Medicine (ACSM) and UK Chief Medical Officers’ guidelines, PA recommendation for adults, including older adults, is for 150 min of moderate activity (55–70% maximum heart rate (HR_max_)/40–60% V̇O_2max_) or 75 min of vigorous activity (70–90% HR_max_/60–85% V̇O_2max_) per week [1, 13, 14]. Though no explicit combination of moderate and vigorous exercise is stated, ACSM recommendations include training sessions of 10–60 min and frequency of 3–5 days∙week^−1^. In recent editions, these recommendations have been supplemented with resistance training 2 days∙week^−1^, and balance and flexibility exercises performed as often as daily [1].

Most exercise interventions for older adults have in the past focussed on a weekly volume of continuous training at moderate or vigorous intensity [1, 14]. High-intensity interval training (HIIT) is also now recognized as an exercise prescription for physical fitness along with continuous intensity training [1, 15]. During a HIIT exercise session, exercise is performed in intervals of submaximal intensity exercise, usually attaining ~ 85–90% HR_max_, interspersed with recovery periods [16]. The alternating work bout and recovery times generally range from 45 s to 4 min [17]. Interval training where work bouts are programmed at supramaximal intensities (greater than the intensity that would achieve V̇O_2max_) is commonly referred to as sprint interval training (SIT) [16, 18]. Using supramaximal intensity usually involves reducing the work bouts to no more than 30 s, with recovery durations lasting several minutes [19]. A further variation, reduced exertion high-intensity training (REHIT) which endeavours to reduce training time by using shorter work bouts and fewer intervals in each session [20], has been studied for effectiveness and acceptance. For the purpose of this review, HIIT will serve as the umbrella term for these types of interval exercise interventions unless otherwise specified.

### Reasons to Evaluate HIIT Effect on Functional Movement

Various iterations of HIIT have been studied, the results showing that in terms of improving cardiorespiratory fitness [21], body composition [22], anaerobic capacity [23] and metabolic health [24], HIIT appears to be at least as effective as MICT. Intervention studies of both healthy [24] and morbid subjects [25, 26] indicate that markers of cardiovascular and metabolic health are significantly improved with exercise prescriptions of either HIIT or MICT, though some ambiguity about the relative effectiveness exists. That said, the evaluation of HIIT interventions can be confounded by the many programming variables, e.g. work and recovery intensity and duration, number of series, frequency, exercise mode, etc. [17]. In comparative studies, the overall volume and intensity of HIIT protocols can be work-matched, iso-caloric, iso-time, or arbitrary relative to MICT interventions, contributing to a lack of consistency and clarity of results.

Research supports HIIT as an effective and time-efficient exercise programme for improving cardiovascular and cardiorespiratory fitness characteristics in older adults, attaining similar results to traditional MICT despite a lower training volume [27]. However, HIIT may have additional effects on the initiation of a traditional exercise intervention on sedentary or untrained older adults [28]. There is evidence that intermittency, as well as intensity, plays a role in the physiological response to exercise training. A three-arm time-matched study incorporating HIIT, MICT, and moderate-intensity interval training (MIIT) on women ≥ 65 years of age (*n* = 43) showed significantly greater effects from MIIT than MICT on body composition and reduction in resting heart rate [29]. This is despite the MICT intervention having higher total work and volume than MIIT. The study further included STS and 6MWT functional movement testing. All three interventions showed significant improvement in the STS, though only HIIT showed significant improvements in 6MWT.

### Change in Muscle Function with Age

Muscle mass, strength and power decline through the ageing process, and neuromuscular function can deteriorate with age through disuse, particularly of type II muscle fibres [30]. In their review of sarcopenia and dynapenia research, Mitchell et al. [31] determined that from a peak in the third decade of life for men and fourth decade of life for women, the mean rate of muscle mass loss is 0.47%∙yr^−1^ for men and 0.37%∙yr^−1^ for women, though in the eighth decade the rate of loss accelerated to 0.8–0.98%∙yr^−1^ and 0.64–0.7%∙yr^−1^, respectively. By age ~ 65 years, muscle power, an important component of functional movement, declines at ~ 3.5%/yr, nearly twice as quickly as strength [32, 33]. Decreases in muscle mass and muscle size of predominantly type II muscle fibres have been associated with increased age [33]. The mechanisms, however, are not fully understood.

### Functional Movement and Muscular Power

Muscle function, particularly muscular power, has a strong association with indicators of functional movement in older adults [31]. Several studies [34, 35] have found that high-velocity resistance training, where rapid or maximum speed concentric activation is used, significantly increases peak torque and average power, along with improvements in measures of functional movement. Both high-velocity resistance training and traditional (using 2–3 s concentric and eccentric contractions) resistance strength training resulted in significant and almost equal (~ 27%) increases in measures of one repetition maximum (1RM) leg press strength in men aged 60–76 years [35]. Both training groups also improved functional movement measures, though only the high-velocity training group achieved statistically significant improvement in the STS and TUG tests.

### High-Intensity Interval Training and Power Adaptations

There is some evidence that HIIT, through excursions into intensities higher than the anaerobic threshold, has an effect on anaerobic capacity. In a randomized controlled trial [27], a 6-week HIIT intervention on healthy older adults aged 65–85 years resulted in significant increases in peak power (159 ± 59 vs. 145 ± 60 W, *p* < 0.001) and anaerobic threshold (15.3 ± 3.8 vs. 13.2 ± 3.4 ml∙kg∙min^−1^, *p* < 0.001) measured using the ramped Bruce protocol cardiopulmonary exercise testing [36]. A related randomized controlled trial [37] showed significant increases in peak power from 2-, 4- and 6-week HIIT interventions in subjects aged 65–85 years. However, at least one parallel study of HIIT and MICT in older adults aged 56–83 years (*n* = 38) has indicated that both interventions can produce significant power adaptations [38], and therefore the relative effectiveness of HIIT in improving measures of power is still unclear.

### Objective

Functional movement is an important factor for the maintenance of health and independence for older adults, though the measurement of functional movement is largely indirect and diverse. While the relationship between muscular power and functional movement is positive and there is evidence of positive power adaptations to HIIT, the variability of HIIT protocols, inconsistency of functional movement testing, and conflicting evidence on the relative efficacy of HIIT and MICT for improving aspects of functional movement indicates a necessity to amalgamate the evidence of functional movement adaptations to HIIT interventions. The objective of this review was to synthesize the existing literature that evaluates the effects of HIIT on functional movement measured by the senior fitness test, SPPB, or components thereof relative to MICT and non-intervention control in adults aged ≥ 50.

## Methods

### Protocol Registration

We searched the Prospero database and Cochrane Library of systematic reviews to ensure that this work was not duplicated by an existing or pending systematic review (SR). A protocol and Prospero registration were submitted and accepted (Prospero ID CRD42021231273) prior to the final search strategy design.

### Definitions and Study Inclusion Criteria

High-intensity interval training was defined as exercise protocols with intermittent high- and low-intensity intervals. The minimum duration of intervention was set at 2 weeks in an effort to capture training adaptations rather than acute effects. No criteria for exercise mode, intensity, work or rest repetition, work or rest time, or frequency were specified in order to incorporate the broadest range of HIIT protocols. Moderate-intensity continuous training was defined as aerobic endurance training at a constant submaximal intensity. No criteria for duration, volume, or intensity of the MICT protocol relative to HIIT were specified.

Functional movement outcome measures were defined as validated measures of functional movement for older adults. These included the SPPB [6], The Senior Fitness Test [7], Functional Movement Screening [3, 9], or components thereof.

Older adults were defined as persons ≥ 50 years old to incorporate a broad spectrum of studies of older adults past their functional capacity peak. No criteria for health status were used, other than the exclusion of neurological impairment or disease to assess physiological adaptation in populations with normally functioning neuromuscular systems and to avoid studies of rehabilitation therapy.

Study inclusion criteria were:

- Randomized controlled and randomized pair-matched intervention studies of HIIT vs non-intervention control, or HIIT vs. MICT.
- Supervised or unsupervised interventions.
- Participants ≥ 50 years of age.
- Primary or secondary outcome measures of functional movement.
- Minimum intervention duration of 2 weeks.

Study exclusion criteria were:

- Studies including children, adolescents, adults < 50 years of age.
- Participants with neurological impairment/disease.
- Studies of combined exercise modality vs control (e.g. HIIT + resistance training).

### Search Strategy

The search strategy was piloted for filters and consistency across databases, then duplicated by an academic librarian and cross-matched. Seven electronic databases: National Library of Medicine (PubMed), Web of Science, SPORTDiscus, Medline, Scopus, Embase, Cumulative Index to Nursing and Allied Health Literature (CINAHL), were searched using appropriate syntax and Boolean operators for each. The search dates were 1 January 1960 to 31 January 2022. Filters were for human studies and an available English language abstract.

Three “gold standard” papers [12, 39, 40] were identified as containing terminology relevant to the search terms and used to cross-check the effectiveness of the search strategy, with each selected database returning at least one of the papers. The search strategy was conducted on each database in four parts: Part 1 was a reference search of terms relating to the intervention (e.g. HIIT, Interval Training). Part 2 was a reference search of terms relating to the primary outcome measure (e.g. mobility, functional movement, physiological functional capacity). Part 3 was a reference search of terms relating to the target population (e.g. older adults, elderly, senior, age 50+). Part 4 was the results from parts one, two and three combined using the Boolean operator AND (see Table 1). Grey literature and dissertation databases were screened for additional references. The results of the seven searches were uploaded into the Covidence Systematic Review Management system (covidence.org).

**Table 1:** Systematic review search terms

Search Term 1 Intervention Search term combined with OR	Search Term 2 Outcome search term combined with OR	Search Term 3 Participant search term combined with OR	Search Term 4 Consolidated search term combined with AND
HIIT	Mobility	(Older adults)	S1
(High-intensity interval training)	(Function* movement)	Elderly	S2
(High-intensity training)	(Function* capacity)	Geriatric*	S3
(Sprint interval training)	(Biomechanic* mobility)	Ageing	
(Interval training)	PFC	Senior	
(Interval exercise)	(Physiologic* functional capacity)	(Older people) (Age 50) (Age 65) (Age 65+)	

### Data Extraction

A data extraction sheet was developed specifically for this systematic review and populated with study data. Extracted data included recruitment and participant information, intervention and comparator information, and pre- and post-intervention outcome measures data. Incomplete data were requested and supplied by study authors [40, 41].

### Risk of Bias and Publication Bias

A risk-of-bias analysis on each study was performed independently by two reviewers. Six domains were assessed: randomization sequence generation, allocation concealment, blinding of assessment, incomplete outcome data, selective reporting, and other bias. In exercise intervention studies, participant blinding is not possible, so that domain was not assessed. Any discrepancies were discussed by all three reviewers until resolved.

Effect size and standard error (see “Meta-analysis” Section) were used to generate funnel plots in the RevMan software [42] and analysed for asymmetry which could indicate publication bias.

### Analysis

#### Meta-analysis

To perform the meta-analysis, the pooled standard deviation and standardized mean difference (SMD) were calculated for each functional movement outcome measure from the individual study data. The formula used was:

$${\text{SMD}} = \frac{{\Delta \overline{x}}}{{\sqrt {\frac{{{\text{SD}}_{1}^{2} + {\text{SD}}_{2}^{2} }}{2}} }}$$

where:

$\Delta \overline{x}$  = within group mean difference.

SD_1_ = standard deviation at baseline.

SD_2_ = standard deviation post-intervention

$$\sqrt {\frac{{{\text{SD}}_{1}^{2} + {\text{SD}}_{2}^{2} }}{2}} = {\text{ pooled}}\;{\text{ standard }}\;{\text{deviation}}$$

The extracted and calculated data were loaded into the RevMan meta-analysis software [42]. The meta-analysis used an inverse variance statistical method on a random effects analysis model to generate the between-groups effect size with 95% confidence interval (CI) comparing the SMD of the HIIT group results with those of the comparator (MICT or control) for each study. The statistical significance threshold was set at *p* = 0.05. Effect sizes were reported as small (0.20), medium (0.50), large (0.80) and very large (1.20) [43].

#### Heterogeneity

Estimates for heterogeneity for each meta-analysis and subgroup were calculated as Tau^2^, the estimate of between-study variance of the group, chi-square, the probability that differences in results are due to chance alone, and *I*^2^, heterogeneity due to between-study variance. Absolute thresholds are not recommended [44]. For the purpose of this review, a chi-square *p* ≤ 0.05 will serve as an indication of heterogeneity, and values of I^2^ have been interpreted as [45]:

- 0–40%: low heterogeneity.
- 30–60%: moderate heterogeneity.
- 50–90%: substantial heterogeneity.
-  > 75%: considerable heterogeneity.

#### Subgroup Analysis

A subgroup analysis of control (CON) as the comparator and MICT as the comparator is required to provide insight into whether the cogent independent variable might be structured exercise activity rather than HIIT in particular. A meta-analysis using a single primary functional movement outcome measure for each study was performed with the chosen outcome measure as described in “Subgroup Analysis –HIIT Versus MICT or Control: Single Outcome for each Study” Section. A second meta-analysis using all functional movement outcome measures from all studies is included. This treats each outcome measure as a separate study with outcome measure weights adjusted by the inverse variance method. Three additional meta-analyses of common outcome measures across studies were performed to evaluate whether the specific outcome measure used substantially altered the results. Meta-analysis of the 10 studies that reported TUG, 12 studies that reported the 6MWT, and nine that reported STS as an outcome measure are presented separately.

Because HIIT protocols for this review include submaximal and supramaximal intensities, it is also interesting to provide subgroup analysis of interventions with interval Intensities > 90% versus < 90% of maximal intensity metric used for each study. This is to evaluate if high to maximal intensity levels would have a different effect than moderate to vigorous intensity intervals [13, 46].

## Results

### Study Selection

**Fig. 1:**
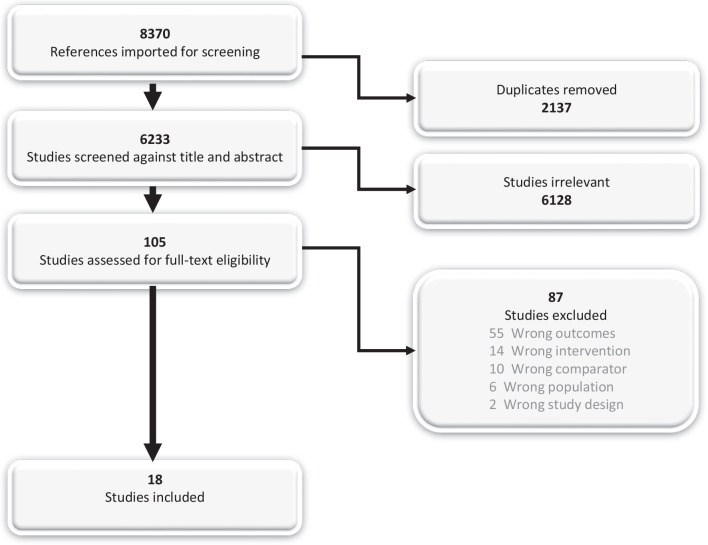
PRISMA flow chart of literature screening for inclusion. From covidence.org

After removing duplicates, the combined searches retrieved 6233 studies. The title and abstract review determined 6128 studies to be irrelevant, leaving 105 studies to be assessed through a full-text review by at least two of the three reviewers. Any disagreements were settled through discussion by the three reviewers. Eighteen studies were ultimately included in the SR. Thereafter, a recursive search and citation search of the included studies reference lists were performed resulting in three additional studies for full-text review by at least two reviewers. None of those studies were included in the SR (Fig. 1).

### Study Characteristics

A total of 851 participants with an average age of 65 ± 6.1 years were recruited and data for 757 participants were included in the studies. The total number of participants completing HIIT interventions was 353 (mean of 18 studies = 19.6; range *n* = 5–60) with 193 completing MICT protocols (mean of 12 studies = 16.1; range *n* = 4–36) and 184 total non-intervention control participants (CON) (mean of eight studies = 23; range *n* = 6–59). Two studies [41, 47] used a three-arm parallel design including MICT and CON. Two studies [48, 49] used a three-arm parallel design including MICT and resistance training comparators.

Two studies [50, 51] did not disclose the sex breakdown of participants; therefore, the sex distribution of the review is not available. Three studies were of female subjects [47, 52, 53], one was of male subjects [53], and 12 studies were of mixed sex [39, 41, 42, 48, 49, 54–60]. Seven studies [39, 41, 47, 52, 53, 56, 60] described participants as untrained. All others did not disclose the level of pre-intervention physical fitness of participants. Pre-intervention PA levels were indicated in four studies [42, 48, 49, 54] as sedentary or insufficiently active. All others did not disclose levels of pre-intervention PA. While all studies were of participants without contraindications to exercise training, five studies [42, 47, 52, 54, 60] reported mixed health conditions and two studies [51, 55] did not disclose participant health status. Three studies [50, 58, 59] were specifically of chronic obstructive pulmonary disease (COPD) patients, two studies [56, 61] were of coronary artery disease patients, and there was one study each of patients with mild-to-moderate Alzheimer’s disease [40], chronic heart failure [56], controlled hypertension [62], and obesity [52]. Two studies [48, 49] described participants as healthy.

Ten studies used cycle ergometers as the training modality [39, 41, 49, 50, 54–59], one used a recumbent bike [41], three used treadmill walking [48, 53, 61], two were non-swimming water-based [52, 60], and one each used dance [46] and land based running [50]. Fifteen studies reported using supervised training sessions[39, 41, 42, 48–50, 52–54, 56–61], one study [50] used an unsupervised intervention, and two studies [47, 55] did not disclose whether the training was supervised or not. Intervention duration averaged 11.75 ± 6.30 weeks with a range of 3–18 weeks. Exercise session length ranged from 11.6 to 60 (mean = 32.66 ± 12.17) minutes for HIIT and 20–60 (mean = 39.22 ± 10.81) minutes for MICT interventions (see Table 2: Study Characteristics).

**Table 2:** Study characteristics

Study identifiers		Participants characteristics				Functional outcome	Comparator		
Author	Ref	#	Average age (years)	Training status	Health status		Intervention comparison	Session (min)	Intensity
﻿Adamson et al.	[62]	17	66	Untrained	Controlled Hypertension	TUG	SIT v Con	NA	NA
Adamson et al.	[54]	12	64.5	ND	ND	TUG, STS	SIT v Con	NA	NA
Ballesta-Garcia et al.	[46]	54	67.8	Untrained	Mixed w/No Contraindications	TUG, 6MWT, STS	HIIT v MICT (iso-time) v Con	60	9–14
Bellumori et al.	[41]	26	70	Untrained	Mixed w/No Contraindications	TUG	HIIT v Con	NA	NA
Bouaziz et al.	[61]	60	73.6	Sedentary	Mixed w/No Contraindications	TUG, 6MWT	HIIT v Con	NA	NA
Boukabous et al.	[52]	18	64.9	Untrained	Obesity	TUG, 6MWT, STS	0.5 time/calorie HIIT vs MICT	50	55% HRR
Coetsee and Terblanche	[47]	67	62.7	Untrained	Healthy	TUG	HIIT v MICT (iso-caloric) v RT v Con	47	70–75% HRmax
Enette et al.	[40]	52	77.9	Untrained	Alzheimer's	6MWT	HIIT v MICT (iso-time) v Con	30	70% HRmax
Gloeckl et al.	[57]	60	53	Untrained	COPD	6MWT	HIIT vs MICT Iso-caloric/Iso-work	10–30	60% PWR
Ikenaga et al.	[50]	81	70.8	Untrained	ND	STS	HIIT v Con	NA	NA
Jaureguizar et al.	[55]	72	58	Untrained	CAD	6MWT	HIIT vs MICT Iso-time	40	VT1—VT1 + 10%
Koufaki et al.	[56]	32	59.1	Untrained	CHF	STS	HIIT v MICT	40	40–60% VO2peak
Mador et al.	[49]	48	72	ND	COPD	6MWT	HIIT vs MICT Iso-work	20	50% Wmax
Nasis et al.	[58]	42	65.5	ND	COPD	6MWT	HIIT vs MICT Iso-work	30	60% Wpeak
Reichert et al.	[59]	36	67.9	Untrained	Mixed w/No Contraindications	TUG, 6MWT, STS	HIIT vs MICT Iso-time	30–36	Borg 13–17
Siqueira-Andrade et al.	[51]	41	64.3	Untrained	Mixed w/No Contraindications	TUG, 6MWT, STS	HIIT vs MICT Iso-time	36	Borg 16
Tavoian et al.	[48]	14	66.4	Untrained	Healthy	6MWT, STS	ca. 0.5 time HIIT v MICT v RT	30–45	50–75% HRR
Wolszakiewicz et al.	[53]	119	58	Untrained	CAD	6MWT	HIIT v Con	NA	NA
		47.3	65.7						

Study identifiers		Interval protocol									
Author	Ref	Exercise Modality	Duration (weeks)	Session (min)	Sessions/ week	Intervals/session	Interval Intensity	Interval (sec)	Recovery Intensity	Recovery (sec)	Time Interval (sec)
﻿Adamson et al.	[62]	Cycle Ergometer	10	11.6	2	10	Max	6	0%	60	NA
Adamson et al.	[54]	Cycle Ergometer	6	ND	2	6–10	Max	6	ND	60	NA
Ballesta-Garcia et al.	[46]	Dance	18	60	2	6–12	Borg 14–18	60–90	Borg 7–11	120–180	NA
Bellumori et al.	[41]	Recumbent Cycle	6	30	2	30	Max	20	Min	40	NA
Bouaziz et al.	[61]	Cycle Ergometer	9.5	30	2	6	VT	240	40%VT	60	NA
Boukabous et al.	[52]	Treadmill walk	8	25	3	6	90% HRR	60	40% HRR	120	NA
Coetsee and Terblanche	[47]	Treadmill walk	16	30	3	4	90–95% HRmax	240	70% HRmax	180	NA
Enette et al.	[40]	Cycle Ergometer	9	30	2	6	80% HRmax	60	60% HRmax	240	NA
Gloeckl et al.	[57]	Cycle Ergometer	3	12–36	5–6		100% PWR	30	0%	30	NA
Ikenaga et al.	[50]	Run	12	189	ND	ND	ND	60	ND	60	NA
Jaureguizar et al.	[55]	Cycle Ergometer	8	40	3	15–30	50% max	20	10% max	40	NA
Koufaki et al.	[56]	Cycle Ergometer	24	30	3	20	100% PPO	30	20–30% PPO	60	NA
Mador et al.	[49]	Cycle Ergometer	8	21	3	7	150% MICT W	60	75% MICT W	120	NA
Nasis et al.	[58]	Cycle Ergometer	10	45	3	40	100% Wpeak prog	30	0%	30	NA
Reichert et al.	[59]	Water-based	28	30–36	2	6-Dec	Borg 15–18	120–240	11–15	30–60	NA
Siqueira-Andrade et al.	[51]	Water-based	12	36	2	18	Borg 18	60	Borg 11	60	NA
Tavoian et al.	[48]	Cycle Ergometer	12	15–30	3	ND	80–100% HRR	15–60	40–60% HRR	15–60	NA
Wolszakiewicz et al.	[53]	Walk	12	36	5–7	6	ND	360	0%	180	NA
			11.75								

*6MWT*  six minute walk test, *CAD*  coronary artery disease, *CHF*  chronic heart failure, *Con*  control, *COPD*  chronic obstructive pulmonary disease, *ND* not disclosed, *HR*_*max*_  heart rate maximum, *HRR*  heart rate reserve, *MICT*  moderate-intensity continuous training, PWR = peak work rate, RT = resistance training, SIT = sprint interval training, *STS*  sit to stand, *TUG*  timed up and go, *V̇O*_*2peak*_  peak rate of oxygen consumption, *VT1*  ventilatory threshold 1, *W*_*max*_  maximum power, *W*_*peak*_  peak power

### Meta-analyses

#### Subgroup Analysis—HIIT Versus MICT or Control: Single Outcome for Each Study

The Short Physical Performance Battery (SPPB) (gait speed, sit to stand, standing balance) [6] or Senior Fitness Test (timed up-and-go, six-minute walk, back scratch, sit and reach, two-minute step, chair stand, arm curl) [7], or components thereof were contained within all 18 included studies. The meta-analysis using a single outcome for each study used the TUG as the primary dependent variable outcome measure where available, as it includes components of level change (rising from and lowering to the seated position), acceleration/deceleration from static start position to static end position, gait speed during the distance walked, and change of direction at the turnaround point. The 6MWT was used for the meta-analysis in seven studies where TUG was not present as it incorporates gait speed and change of direction. The STS was used for the meta-analysis in two studies where TUG and 6MWT were not present.

##### HIIT Versus MICT

**Fig. 2:**
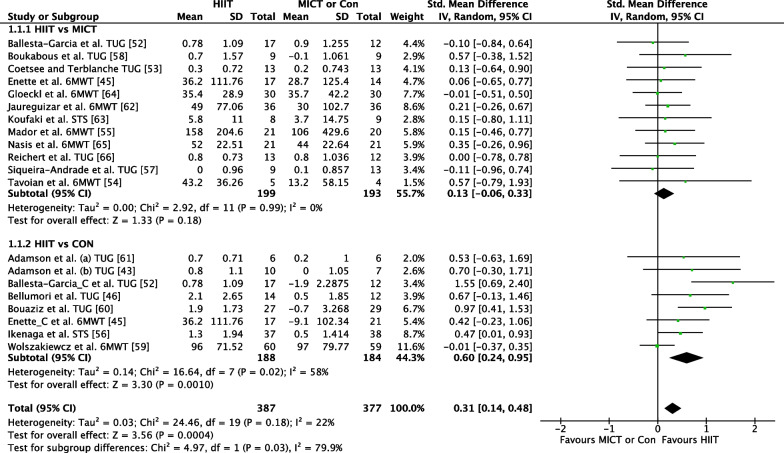
Meta-analysis subgroups HIIT versus MICT, HIIT versus Control with single outcome for each study

The effect of HIIT vs. MICT on functional movement outcomes was investigated in 12 studies (*n* = 391) [41, 47–50, 52, 53, 56, 58–60]. Figure 2 shows a trivial non-significant effect (ES = 0.13 95% CI [−0.06, 0.33] *p* = 0.18). No heterogeneity was evident.

##### HIIT Versus CON

The effect of HIIT vs. CON on functional movement outcomes was investigated in eight studies (*n* = 338) [44, 46, 47, 53, 57, 60, 61, 62,]. Figure 2 shows a significant effect in favour of HIIT (ES = 0.60 95% CI [0.24, 0.95] *p* = 0.001). Moderate heterogeneity was evident, with three of eight studies reporting significant findings in favour of HIIT.

#### HIIT *Versus* MICT or Control: All Functional Movement Outcome Measures Treated as Separate Studies

##### HIIT Versus MICT

**Fig. 3:**
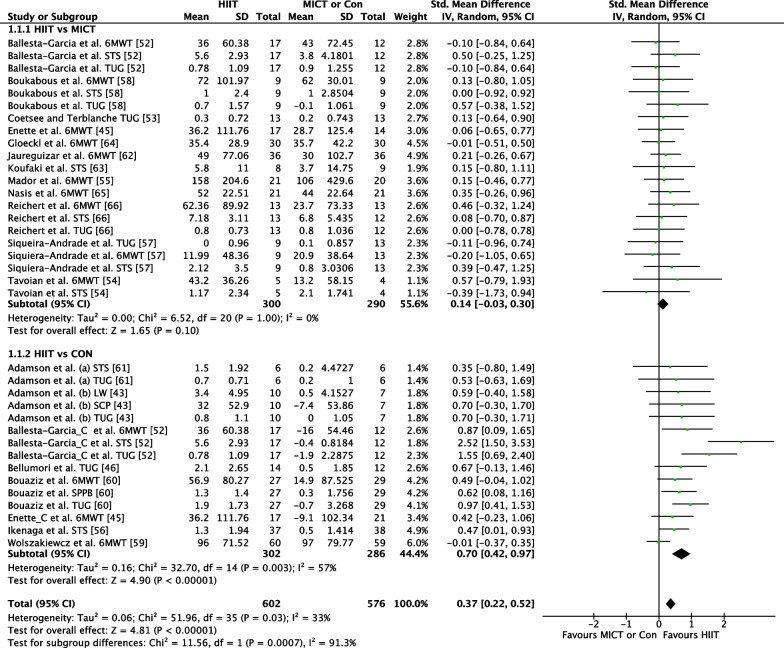
Meta-analysis subgroups HIIT versus MICT, HIIT versus Control with all functional movement outcome measures for each study

The effect of HIIT vs. MICT on functional movement outcomes was investigated in 12 studies (*n* = 391) reporting 21 outcome measures [41, 47–50, 52, 53, 56–60]. Figure 3 shows a trivial non-significant effect (ES = 0.14 95% CI [−0.03, 0.30] *p* = 0.10). No heterogeneity was evident.

##### HIIT Versus CON

The effect of HIIT vs. CON on functional movement outcomes was investigated in eight studies (*n* = 338) reporting 15 outcome measures [39, 41, 42, 47, 51, 54, 55, 61]. Figure 3 shows a medium significant effect in favour of HIIT (ES = 0.70 95% CI [0.42, 0.97] *p* < 0.001). Low heterogeneity was evident, with six of 15 outcome measures reporting significant findings in favour of HIIT.

#### HIIT *Versus* MICT or Control: Outcome 6MWT

##### HIIT Versus MICT

**Fig. 4:**
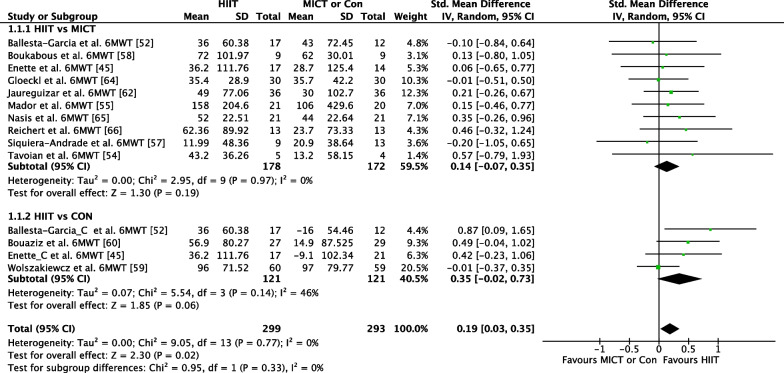
Meta-analysis subgroups HIIT versus MICT, HIIT versus Control with 6MWT outcome measure

The effect of HIIT versus MICT on the 6MWT functional movement outcome was investigated in 10 studies (*n* = 349) [41, 47, 49, 50, 52, 53, 56, 58–60]. Figure 4 shows a trivial non-significant effect (ES = 0.14 95% CI [−0.07, 0.35] *p* = 0.19). No heterogeneity was evident.

##### *HIIT *Versus* CON*

The effect of HIIT vs. CON on the 6MWT functional movement outcome was investigated in four studies (*n* = 221) [41, 47, 54, 61]. Figure 4 shows a small effect in favour of HIIT that approached significance (ES = 0.35 95% CI [−0.02, 0.73] *p* = 0.06). Moderate heterogeneity was evident, with one of four studies reporting significant findings in favour of HIIT.

#### HIIT Versus MICT or Control: Outcome TUG

##### *HIIT *Versus* MICT*

**Fig. 5:**
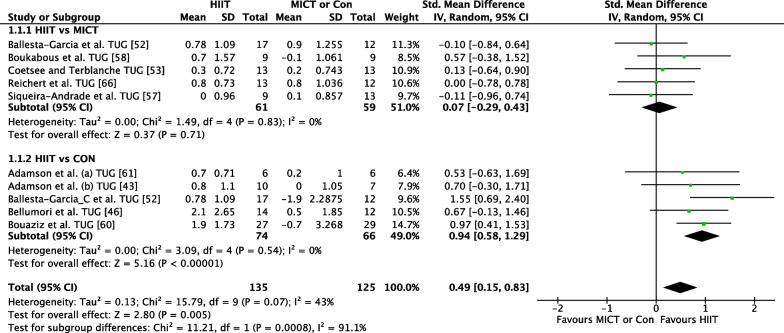
Meta-analysis subgroups HIIT versus MICT, HIIT versus Control with TUG outcome measure

The effect of HIIT versus MICT on the TUG functional movement outcome was investigated in five studies (*n* = 120) [47, 48, 52, 53, 60]. Figure 5 shows a trivial non-significant effect (ES = 0.07 95% CI [−0.29, 0.43] *p* = 0.71). No heterogeneity was evident.

##### *HIIT *Versus* CON*

The effect of HIIT vs. CON on the TUG functional movement outcome was investigated in five studies (*n* = 140) [39, 42, 47, 54, 55]. Figure 5 shows a large significant effect in favour of HIIT (ES = 0.94 95% CI [0.58, 1.29] *p* < 0.001). No heterogeneity was evident, with two of four studies reporting significant findings in favour of HIIT.

#### HIIT Versus MICT or Control: Outcome STS

##### *HIIT *Versus* MICT*

**Fig. 6:**
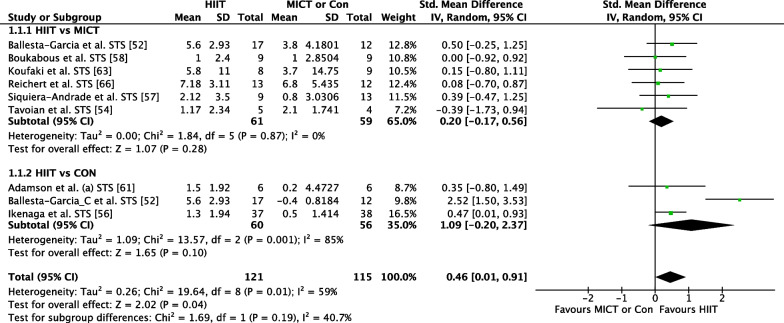
Meta-analysis subgroups HIIT versus MICT, HIIT versus Control with STS outcome measure

The effect of HIIT versus MICT on the STS functional movement outcome was investigated in six studies (*n* = 120) [47, 49, 52, 53, 57, 60]. Figure 6 shows a small non-significant effect (ES = 0.20 95% CI [−0.17, 0.56] *p* = 0.28). No heterogeneity was evident.

##### *HIIT *Versus* CON*

The effect of HIIT versus CON on the STS functional movement outcome was investigated in three studies (*n* = 116) [47, 51, 55]. Figure 6 shows a large non-significant effect (based on significance criterion of *p* = 0.05) in favour of HIIT (ES = 1.09 95% CI [−0.20, 2.37] *p* = 0.10). Substantial heterogeneity was evident, with two of three studies reporting significant findings in favour of HIIT.

#### HIIT Versus MICT or Control: Subgroups Intensity

##### *HIIT *Versus* MICT* Intensity > 90%

**Fig. 7:**
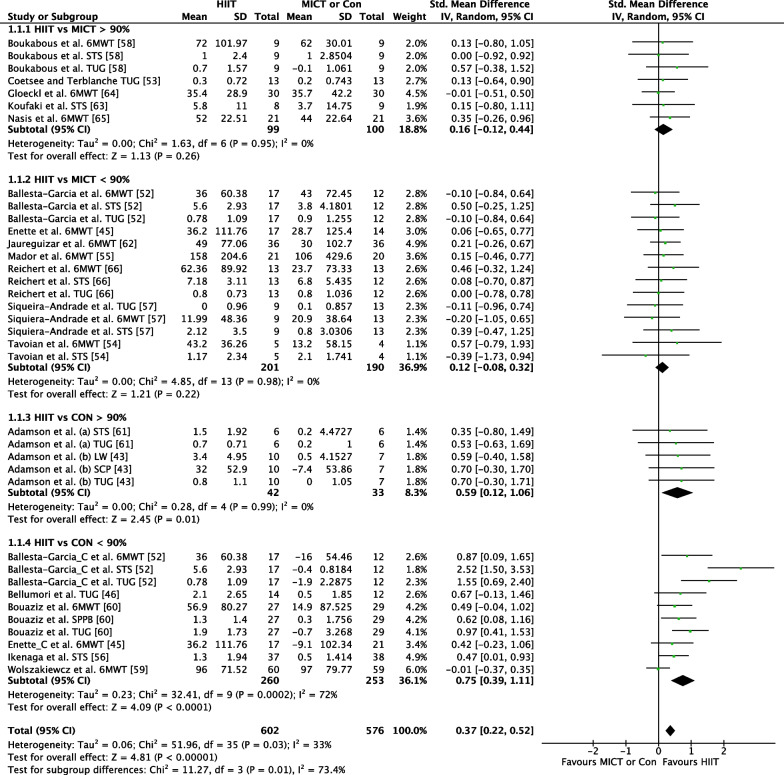
Meta-analysis subgroups HIIT versus MICT, HIIT versus Control with subgroups > 90% HIIT interval intensity and < 90% HIIT interval intensity with all functional movement outcome measures for each study

The effect of HIIT intensities ≥ 90% of maximum vs. MICT on functional movement outcomes was investigated in five studies (*n* = 163) reporting seven outcomes [6, 48, 53–59, 61]. Figure 7 shows a trivial non-significant effect (ES = 0.16 95% CI [−0.12, 0.44] *p* = 0.26). No heterogeneity was evident.

##### *HIIT *Versus* MICT* Intensity < 90%

The effect of HIIT intensities < 90% of maximum vs. MICT on functional movement outcomes was investigated in seven studies (*n* = 224) reporting 14 outcomes [41, 47, 49, 50, 52, 56, 60]. Figure 7 shows a trivial non-significant effect (ES = 0.12 95% CI [−0.08, 0.32] *p* = 0.22). No heterogeneity was evident.

##### *HIIT *Versus* CON* Intensity > 90%

The effect of HIIT intensities ≥ 90% of maximum versus CON on functional movement outcomes was investigated in two studies (*n* = 29) reporting five outcomes [39, 55]. Figure 7 shows a medium significant effect in favour of HIIT (ES = 0.59 95% CI [0.12, 1.06] *p* = 0.01). No heterogeneity was evident.

##### *HIIT *Versus* CON* Intensity < 90%

The effect of HIIT intensities < 90% of maximum vs. CON on functional movement outcomes was investigated in six studies (*n* = 322) reporting 10 outcomes [41, 42, 47, 51, 54, 61]. Figure 7 shows a medium significant effect in favour of HIIT (ES = 0.75 95% CI [0.39, 1.11] *p* < 0.001). Substantial heterogeneity was evident with six of 10 outcomes reporting significant findings in favour of HIIT.

### Risk-of-Bias Analysis

**Fig. 8:**
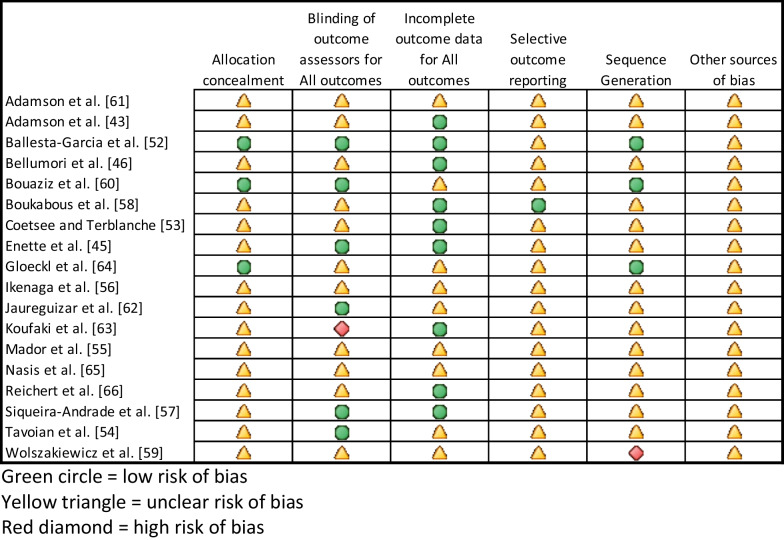
Risk-of-bias summary. Green circle = low risk of bias, Yellow triangle = unclear risk of bias, Red diamond = high risk of bias

Three of the studies [47, 54, 58] included specific details of the randomization sequence generation and process. Gloeckl et al., [58], Ballesta-García et al*.* [47], Bouaziz et al*.* [54] also indicated a specifically concealed allocation process. Four studies [41, 47, 52, 54] unambiguously indicated assessor blinding, while one study [56] was explicitly not assessor blinded. Eight studies were assessed as having complete information on all participants recruited, participant attrition, and participants completed (Fig. 8).

### Publication Bias Analysis

**Fig. 9:**
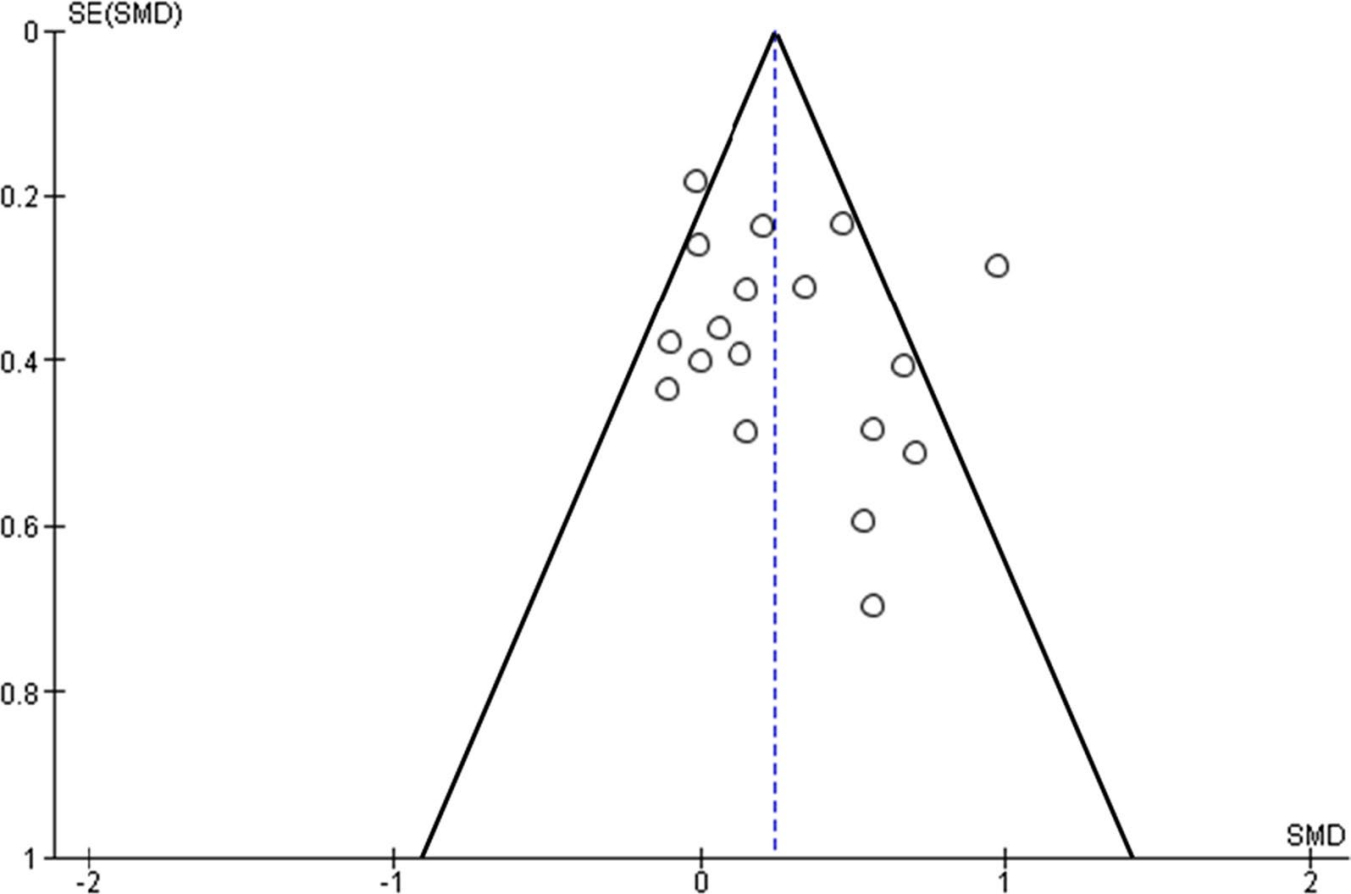
Effect size and standard error funnel plot

A funnel plot of effect size and standard error (Fig. 9) indicated a fairly symmetrical distribution with eight studies above, nine studies below and one study approximately at the standardized mean difference. This distribution would not indicate a publication or small study bias.

## Discussion

The purpose of this review was to evaluate evidence of changes in functional movement in older adults due to exercise training with HIIT versus MICT or non-intervention (CON). Versus CON, HIIT is an effective mode of exercise for improving functional movement in older adults. However, functional movement improvements were not significantly different between HIIT and MICT.

### HIIT Versus CON

We found significant and small (0.35) to large (1.09) positive effects of HIIT vs. CON on almost all functional movement outcomes. Only the subgroup analysis of STS had a statistically non-significant outcome, though this subgroup had only three studies, which likely also accounts for the statistical heterogeneity of that analysis.

As with the initiation of most forms of exercise, the results showing that HIIT interventions improve measures of functional movement are consistent with systematic reviews of HIIT studies in older adults measuring other physiological variables [60]. In a systematic review and meta-analysis of HIIT effects on blood pressure, researchers found large and significant mean improvement in both diastolic and systolic blood pressure from 10 studies of adults aged ≥ 60 years [63]. Similarly, significant improvements in peak oxygen uptake (V̇O_2peak_) were found following HIIT versus CON in adults aged ≥ 65 years [64].

Subgroup analysis by higher (> 90% of maximal intensity metric) and lower HIIT exercise intensity produced similar results to each other. Though both subgroups of higher- and lower-intensity HIIT versus CON resulted in medium effect size and were statistically significant, one limitation is that there are only two underlying studies [39, 55] categorized as higher intensity. In a systematic review of HIIT effects on body composition [65] researchers used a similar subgroup analysis of high-intensity (> 90% peak heart rate) and low-intensity (< 90% peak heart rate) interventions. This analysis, however, had a mean participant age of 38.8 ± 14.4 years which might limit comparisons with the present review. Higher-intensity protocols are less often available in studies of older adults and could be an area for future research.

### HIIT Versus MICT

The meta-analysis of HIIT vs. MICT showed trivial effects in each of the subgroup analyses. However, several variations in the HIIT and MICT protocols may have created ambiguity in the results. Five of the studies [47, 49, 52, 53, 60] had multiple functional movement outcome measures (Fig. 3) and interestingly, within each of those studies the outcome measures did not show agreement in favour of HIIT or MICT. One study [59] showed a small effect in favour of HIIT in the 6MWT, but trivial effects in the TUG and STS which may have been due to the HIIT intensity level using a rate of perceived exertion (RPE) 15–18 [66] and MICT intensity level of RPE 13–17, both reaching the vigorous category [13] and therefore perhaps too small of an intensity differentiation between protocols. Results in the study by Ballesta-García et al*.* [47] showed a medium effect in favour of HIIT in the STS test, but trivial effects in the 6MWT and TUG. One study [52] indicated a medium effect size favouring HIIT in TUG, but trivial effects for 6MWT and STS using a HIIT exercise session duration of exactly half that of MICT, while another study using iso-time protocols [51] reported a small effect in favour of HIIT in STS, but a small effect in favour of MICT in 6MWT. The study by Tavoian et al*.* [48] showed a medium effect in favour of HIIT for the 6MWT but a small effect in favour of MICT for the STS. It should also be mentioned that no single outcome measure of HIIT versus MICT in any of the included studies reached statistical significance.

There was no significant difference in functional movement outcomes between MICT and HIIT carried out at > 90% or < 90% of maximum intensity. There are two potential limitations to interpreting these results. Firstly, the intensity regulation of the HIIT subgroups was inconsistent. In the higher-intensity subgroup, one study [53] used an interval intensity of 90% of heart rate reserve (HRR); one [48] used intervals achieving 90–95% of HR_max_; and three used interval intensities of 100% of pre-intervention incremental exercise tests of peak work rate [58], or peak power output [57, 59]. In the lower-intensity subgroup, three studies used RPE up to 18 [47, 52, 60], one used 80% of HRR [48], another used 80% of HR_max_ [40]. One [55] used a work bout intensity of 50% of pretesting maximum power, and one [49] used 75%. Secondly, variations in the comparative MICT protocol were evident. Five studies used iso-time MICT to HIIT [41, 47, 52, 56, 60]. Two studies used a MICT protocol of twice the training time to that of the HIIT group [49, 53], two used iso-caloric MICT and HIIT protocols [48, 58], two used an iso-work MICT to HIIT comparator [50, 59] and one was unspecified [56]. Protocols comparing HIIT and MICT may sometimes be iso-time, though more often than not HIIT will have a lower total work and calorie expenditure than MICT, with these parameters not even clearly documented between studies, contributing to a lack of comparability. Variability in protocol and study characteristics have been recognized in other systematic reviews of HIIT versus MICT [67–69]. Given the potential differences in protocol intensities and programming, there is some debate about whether equalization by energy expenditure or workload is necessary for comparative studies [69], though some researchers argue that research on HIIT as an alternative to MICT should focus on physiological adaptation at unequal volume or energy expenditure [70].

The range of intervention duration of 3–28 weeks may be another confounding factor in the present review. Only four of the studies [47, 48, 57, 60] had intervention durations > 12 weeks. Those studies had some of the smallest between-group effect sizes versus MICT, indicating no significant relative effect of longer-duration interventions of HIIT. However, due to the relatively small number of studies and diversity of programming, these results might be seen as inconclusive.

Researchers have found that in active healthy subjects there is a significant dose–response effect for increases in either exercise volume or intensity [71]. There is good evidence for a dose–response effect for PA in older adults [72] with increasing levels of PA associated with progressively lower mortality risk. A meta-analysis of HIIT interventions in older adults [73] measuring flow-mediated dilation as an indicator of cardiovascular disease reported a significant effect from ≥ 8 weeks of vigorous intensity, but no statistically significant effect from ≤ 8 weeks of moderate-intensity exercise. The clinical training effect of the high- and low-intensity subgroups had similar values, though statistical significance was not achieved by the lower-intensity group. Noteworthy is that this meta-analysis had only nine underlying studies, one of which [61] is included in the present analysis. Illustrating the difficulty of categorization, this meta-analysis considered the study [61] to be in the higher-intensity group due to the HIIT work bouts being above the moderate intensity level, while the present analysis put it in the < 90% of maximum intensity subgroup analysis. Overall, it is possible that the inconsistency of intensity regulation, broad range of intervention duration, and small number of underlying studies of the present review are confounding factors in the higher- versus lower-intensity subgroup analysis.

### Pre-training Status

Baseline fitness has also been shown to be an important covariate of physiological adaptation to exercise training [74]. However, there is some evidence that the HIIT modality may incorporate mechanisms not intrinsic in other forms of exercise. Knowles et al*.* [29] observed similar adaptations to HIIT interventions for V̇O_2max_ in lifelong exercisers (12.5%) and sedentary subjects (11.0%). This is in contrast to other studies that indicate a negative correlation between physiological adaptation to HIIT or MICT and baseline fitness level [21]. In the present review, pre-intervention training status was indicated in 15/18 studies as simply untrained or sedentary, with three studies [50, 55, 59] not reporting. Pre-intervention health status of participants was diverse, including three studies [50, 58, 59] of subjects with chronic obstructive pulmonary disease (COPD), two studies [56, 61] of coronary artery disease (CAD), and one each of Alzheimer’s disease [40] and coronary heart failure (CHF) [56] patients. The response to the initiation of an exercise regime in untrained populations with differential health status may have overwhelmed any apparent differentiation between HIIT and MICT interventions. More data on the effects of HIIT on healthy and trained populations would be necessary to confirm this.

### HIIT and Power Adaptations

Increases in absolute and relative PPO have been observed in studies of HIIT in masters athletes [75] and sedentary subjects [76]. The positive association between power and functional movement has been understood for some time [36, 77], and power training has also been shown to be effective for elderly populations, with significant increases in power associated with significant improvement in functional movement in frail care facility residents [35, 78] as well as healthy community-dwelling older adults [39]. Researchers have also observed that even in elderly, mobility-limited subjects, power training resulted in increased gait speed which was attributed to improvements in voluntary muscle activation [79]. This is in contrast to endurance strength training of two or more resistance exercise pairs performed at moderate intensity with short recovery periods, which has been shown to be ineffective at improving functional movement [80]. However, the HIIT versus MICT subgroup analysis in the present review resulted in only trivial between-group effects (ES = 0.13 95% CI [−0.06, 0.33] *p* = 0.18). The speculation that HIIT replicates aspects of power training is not unambiguously supported by the results, though the differences in HIIT versus MICT protocol characteristics in the present analysis may not have been conducive to power adaptations.

### Functional Movement Measurement of Older Adults

The short physical performance battery (SPPB) was developed to assess lower extremity function in frail and pre-frail elderly [6]. The tests use no upper body or transverse plane movements. Rikli and Jones [81] developed the senior fitness test (SFT) to evaluate functional fitness and subsequently to establish criteria-based fitness standards that might predict the ability of the elderly to maintain independence [8]. The SFT includes an arm curl exercise and an upper shoulder mobility assessment, but these movements serve as indicators rather than direct tests of upper body functionality [7]. Transverse and frontal plane movements are absent also from the SFT. In principle, these tests are held up as assessments of functional ability. In practice, however, the TUG, 6MWT and STS are all timed trials with no systematic evaluation of movement patterns. Indicators of lateral imbalances or compensation strategies are not incorporated into these assessments.

Though none of the included studies used the Functional Movement Screening (FMS) [3], the test was developed as an evaluation of mobility and stability through seven upper, lower and whole body movements designed to identify imbalances and asymmetries. There are studies that have used the FMS to evaluate older populations, and a study of 583 participants aged ≥ 55 years found the highest correlations with waist circumference (*r* = −0.43) and PA (*r* = 0.42) [12]. The present review would have benefitted from studies using a more complete evaluation of functional movement similar to the FMS.

## Conclusion

This is the first systematic review and meta-analysis to amalgamate the current research on the effects of HIIT vs. MICT and CON on functional movement in older adults. The results of the various subgroup meta-analyses confirm that HIIT is similarly beneficial to MICT as an effective exercise regime for improvements in standard measures of functional movement in untrained older adults. However, while the results of the present meta-analysis indicate very low statistical heterogeneity of results, narrative analysis shows that the study characteristics are highly varied. The inclusion of cohorts with various morbidities and the broad range of HIIT protocols compound the complexity of analysing the effects of HIIT. To isolate the relative effects of HIIT as it relates to functional movement in older adults, future research should utilize more standardized HIIT protocols, equalized to the comparator for work, time, or energy expenditure, include HIIT interventions on healthy, physically active populations, and measure functional movement with more comprehensive assessments than those designed to assess the frail elderly.

## Data Availability

The data sets generated during and/or analysed during the current study are available from the corresponding author upon reasonable request.

## Abbreviations

6MWT: Six-minute walk test
ACSM: American College of Sports Medicine
CAD: Coronary artery disease
CHF: Chronic heart failure
CI: Confidence interval
CON: Control
COPD: Chronic obstructive pulmonary disease
ES: Effect size
FMS: Functional movement screening
HIIT: High-intensity interval training
HR_max_: Heart rate maximum
HRR: Heart rate reserve
MICT: Moderate-intensity continuous training
MIIT: Moderate-intensity interval training
PA: Physical activity
PFC: Physiological functional capacity
PPO: Peak power output
PWR: Peak work rate
RPE: Rate of perceived exertion
RT: Resistance training
SIT: Sprint interval training
SMD: Standardized mean difference
SPPB: Short physical performance battery
STS: Chair sit-to-stand
TUG: Timed up and go
V̇O_2max_: Maximum rate of oxygen consumption
V̇O_2peak_: Peak rate of oxygen consumption
VT1: Ventilatory threshold 1
*W*_max_: Maximum power
*W*_peak_: Peak power

## References

[1] ACSM, ACSM’s guidelines for exercise testing and prescription, Eleventh. Wolters Kluwer, 2021.

[2] Rose DJ, Physical activity instruction of older adults. Human Kinetics, 2019.

[3] Cook G, Burton L, Hoogenboom BJ, Voight M (2014). Functional movement screening: the use of fundamental movements as an assessment of function - part 2. Int J Sport Phys Ther.

[4] Tanaka H, Seals DR (1997). Age and gender interactions in physiological functional capacity: insight from swimming performance. J Appl Physiol.

[5] Donato AJ, Tench K, Glueck DH, Seals DR, Eskurza I, Tanaka H (2003). Declines in physiological functional capacity with age: a longitudinal study in peak swimming performance. J Appl Physiol.

[6] Guralnik JM (1994). A short physical performance battery assessing lower extremity function: association with self-reported disability and prediction of mortality and nursing home admission. J Gerontol.

[7] Rikli RE, Jones CJ (1999). Development and validation of a functional fitness test for community-residing older adults. J Aging Phys Act.

[8] Rikl R, Jones C (2013). Development and validation of criterion-referenced clinically relevant fitness standards for maintaining physical independence in later years. Gerontologist.

[9] Cook G, Burton L, Hoogenboom BJ, Voight M (2014). Functional movement screening: the use of fundamental movements as an assessment of function - part 1. Int J Sports Phys Ther.

[10] Perry FT, Koehle MS (2013). Normative data for the functional movement screen in middle-aged adults. J Strength Cond Res.

[11] Mitchell UH, Johnson AW, Vehrs PR, Feland JB, Hilton SC (2016). Performance on the functional movement screen in older active adults. J Sport Heal Sci.

[12] Farrell SW (2019). Functional movement screening performance and association with key health markers in older adults. J Strength Cond Res.

[13] Norton K, Norton L, Sadgrove D (2010). Position statement on physical activity and exercise intensity terminology. J Sci Med Sport.

[14] Davies DSC, Atherton F, McBride M, and Calderwood C, UK chief medical officers’ physical activity guidelines, Dep Heal Soc Care, 2019; pp. 1–65.

[15] Wen D (2019). Effects of different protocols of high intensity interval training for VO2max improvements in adults: a meta-analysis of randomised controlled trials. J Sci Med Sport.

[16] Weston KS, Wisløff U, Coombes JS (2014). High-intensity interval training in patients with lifestyle-induced cardiometabolic disease: a systematic review and meta-analysis. Br J Sports Med.

[17] Buchheit M, Laursen PB (2013). High-intensity interval training, solutions to the programming puzzle: part I: cardiopulmonary emphasis. Sports Med.

[18] MacInnis MJ, Gibala MJ (2017). Physiological adaptations to interval training and the role of exercise intensity. J Physiol.

[19] Gibala MJ (2006). Short-term sprint interval versus traditional endurance training: similar initial adaptations in human skeletal muscle and exercise performance. J Physiol.

[20] Metcalfe RS, Babraj JA, Fawkner SG, Vollaard NBJ (2012). Towards the minimal amount of exercise for improving metabolic health: beneficial effects of reduced-exertion high-intensity interval training. Eur J Appl Physiol.

[21] Milanović Z, Sporiš G, Weston M (2015). Effectiveness of high-intensity interval training (HIT) and continuous endurance training for VO2max improvements: a systematic review and meta-analysis of controlled trials. Sports Med.

[22] Shepherd SO (2015). Low-volume high-intensity interval training in a gym setting improves cardio-metabolic and psychological health. PLoS ONE.

[23] Little JP, Francois ME (2014). High-intensity interval training for improving postprandial hyperglycemia. Res Q Exerc Sport.

[24] Gibala M, Little J, Macdonald M, Hawley J (2012). Physiological adaptations to low-volume, high-intensity interval training in health and disease. J Physiol.

[25] Moholdt TT (2009). Aerobic interval training versus continuous moderate exercise after coronary artery bypass surgery: a randomized study of cardiovascular effects and quality of life. Am Heart J.

[26] de Sant’Ana LO (2020). Effects of cardiovascular interval training in healthy elderly subjects: a systematic review. Front Physiol.

[27] Herrod PJJ, Atherton PJ, Smith K, Williams JP, Lund JN, Phillips BE (2021). Six weeks of high-intensity interval training enhances contractile activity induced vascular reactivity and skeletal muscle perfusion in older adults. GeroScience.

[28] Knowles AM, Herbert P, Easton C, Sculthorpe N, Grace FM (2015). Impact of low-volume, high-intensity interval training on maximal aerobic capacity, health-related quality of life and motivation to exercise in ageing men. Age (Omaha).

[29] Coswig VS, Barbalho M, Raiol R, Del Vecchio FB, Ramirez-Campillo R, Gentil P (2020). Effects of high vs moderate-intensity intermittent training on functionality, resting heart rate and blood pressure of elderly women. J Transl Med.

[30] Lexell J (1995). Human aging, muscle mass, and fiber type composition. J Gerontol A Biol Sci Med Sci.

[31] Skelton D, Greig C, Davies J, Young A (1994). Strength, power and related functional ability of healthy people aged 65–89 years. Age Ageing.

[32] Metter EJ, Conwit R, Tobin J, Fozard JL (1997). Age-associated loss of power and strength in the upper extremities in women and men. J Gerontol Ser A Biol Sci Med Sci.

[33] Lexell J, Taylor CC, Sjöström M (1988). What is the cause of the ageing atrophy?. Total number, size and proportion of different fiber types studied in whole vastus lateralis muscle from 15- to 83-year-old men. J Neurol Sci.

[34] Hruda KV, Hicks AL, McCartney N (2003). Training for muscle power in older adults: effects on functional abilities. Can J Appl Physiol.

[35] Bottaro M, Machado SN, Nogueira W, Scales R, Veloso J (2007). Effect of high versus low-velocity resistance training on muscular fitness and functional performance in older men. Eur J Appl Physiol.

[36] Will PM, Walter JD (1999). Exercise testing: improving performance with a ramped Bruce protocol. Am Heart J.

[37] Herrod PJJ (2020). The time course of physiological adaptations to high-intensity interval training in older adults. Aging Med.

[38] O’brien MW, Johns JA, Robinson SA, Bungay A, Mekary S, Kimmerly DS (2020). Impact of high-intensity interval training, moderate-intensity continuous training, and resistance training on endothelial function in older adults. Med Sci Sport Exerc.

[39] García-Pinillos F, Laredo-Aguilera JA, Muñoz-Jiménez M, Latorre-Román PA (2019). Effects of 12-week concurrent high-intensity interval strength and endurance training program on physical performance in healthy older people. J Strength Cond Res.

[40] Enette L (2020). Effect of 9 weeks continuous vs. interval aerobic training on plasma BDNF levels, aerobic fitness, cognitive capacity and quality of life among seniors with mild to moderate Alzheimer’s disease: a randomized controlled trial. Eur Rev Aging Phys Act.

[41] Bellumori M, Uygur M, Knight CA (2017). High-speed cycling intervention improves rate-dependent mobility in older adults. Med Sci Sports Exerc.

[42] Review Manager 5.4.1. The cochrane collaboration, 2020.

[43] Cohen J (1992). A power primer. Psychol Bull.

[44] Higgins J et al. (Eds), Cochrane Handbook for Systematic Reviews of Interventions version 6.2. Cochrane, 2021.

[45] Higgins J et al. (Eds), Cochrane Handbook for Systematic Reviews of Interventions, 6.3. Cochrane, 2022.

[46] Ballesta-García I, Martínez-González-Moro I, Rubio-Arias J, Carrasco-Poyatos M (2019). High-intensity interval circuit training versus moderate-intensity continuous training on functional ability and body mass index in middle-aged and older women: a randomized controlled trial. Int J Environ Res Publ Health.

[47] Coetsee C, Terblanche E (2017). The effect of three different exercise training modalities on cognitive and physical function in a healthy older population. Eur Rev Aging Phys Act.

[48] Tavoian D (2021). Effects of three different exercise strategies for optimizing aerobic capacity and skeletal muscle performance in older adults: a pilot study. J Frailty Aging.

[49] Mador M, Krawz A, Alhajhusian A, Khan A, Shaffer M, Kufel T (2009). Interval training versus continuous training in patients with chronic obstructive pulmonary disease. J Cardiopulm Rehabil Prev.

[50] Ikenaga M (2016). Effects of a 12-week, short-interval, intermittent, low-intensity, slow-jogging program on skeletal muscle, fat infiltration, and fitness in older adults: randomized controlled trial. Eur J Appl Physiol.

[51] Siqueira-Andrade L (2020). Randomized clinical trial of water-based aerobic training in older women (WATER Study): functional capacity and quality of life outcomes. J Phys Act Heal.

[52] Boukabous I (2019). Low-volume high-intensity interval training (HIIT) versus moderate-intensity continuous training on body composition, cardiometabolic profile and physical capacity in older women. J Aging Phys Act.

[53] Wolszakiewicz J, Piotrowicz E, Foss-Nieradko B, Dobraszkiewicz-Wasilewska B, Piotrowicz R (2015). A novel model of exercise walking training in patients after coronary artery bypass grafting. Kardiol Pol.

[54] Adamson SB, Lorimer R, Cobley JN, Babraj JA (2014). Extremely short-duration high-intensity training substantially improves the physical function and self-reported health status of elderly adults. J Am Geriatr Soc.

[55] Jaureguizar K (2016). Effect of high-intensity interval versus continuous exercise training on functional capacity and quality of life in patients with coronary artery disease: a randomized clinical trial. J Cardiopulm Rehabil Prev.

[56] Koufaki P, Mercer T, George K, Nolan J (2014). Low-volume high-intensity interval training vs continuous aerobic cycling in patients with chronic heart failure: a pragmatic randomised clinical trial of feasibility and effectiveness. J Rehabil Med.

[57] Gloeckl R, Halle M, Kenn K (2012). Interval versus continuous training in lung transplant candidates: a randomized trial. J Heart Lung Transpl.

[58] Nasis IG (2009). Effects of interval-load versus constant-load training on the BODE index in COPD patients. Respir Med.

[59] Reichert T, Kanitz AC, Delevatti RS, Bagatini NC, Barroso BM, Kruel LFM (2016). Continuous and interval training programs using deep water running improves functional fitness and blood pressure in the older adults. Age.

[60] Wu Z-J, Wang Z-Y, Gao H-E, Zhou X-F, Li F-H (2021). Impact of high-intensity interval training on cardiorespiratory fitness, body composition, physical fitness, and metabolic parameters in older adults: a meta-analysis of randomized controlled trials. Exp Gerontol.

[61] Bouaziz W (2019). Effects of a short-term interval aerobic training programme with active recovery bouts (IATP-R) on cognitive and mental health, functional performance and quality of life: a randomised controlled trial in sedentary seniors. Int J Clin Pract.

[62] Adamson S, Kavaliauskas M, Yamagishi T, Phillips S, Lorimer R, Babraj J (2019). Extremely short duration sprint interval training improves vascular health in older adults. Sport Sci Health.

[63] Carpes L, Costa R, Schaarschmidt B, Reichert T, Ferrari R (2022). High-intensity interval training reduces blood pressure in older adults: a systematic review and meta-analysis. Exp Gerontol.

[64] Bouaziz W, Malgoyre A, Schmitt E, Lang PO, Vogel T, Kanagaratnam L (2020). Effect of high-intensity interval training and continuous endurance training on peak oxygen uptake among seniors aged 65 or older: a meta-analysis of randomized controlled trials. Int J Clin Pract.

[65] Maillard F, Pereira B, Boisseau N (2018). Effect of high-intensity interval training on total, abdominal and visceral fat mass: a meta-analysis. Sports Med.

[66] Borg GAV (1982). Psychophysical bases of perceived exertion. Med Sci Sport Exerc.

[67] Hannan A (2018). High-intensity interval training versus moderate-intensity continuous training within cardiac rehabilitation: a systematic review and meta-analysis. Open Access J Sport Med.

[68] Wen D (2019). Effects of different protocols of high intensity interval training for VO 2 max improvements in adults: a meta-analysis of randomised controlled trials. J Sci Med Sport.

[69] Andreato LV (2020). High-intensity interval training: methodological considerations for interpreting results and conducting research. Trends Endocrinol Metab.

[70] Vollaard NBJ, Metcalfe RS (2021). Those apples don’t taste like oranges! Why ‘Equalising’ HIIT and MICT protocols does not make sense. Trends Endocrinol Metab.

[71] Foulds HJA, Bredin SSD, Charlesworth SA, Ivey AC, Warburton DER (2014). Exercise volume and intensity: a dose-response relationship with health benefits. Eur J Appl Physiol.

[72] Ekelund U et al., Dose-response associations between accelerometry measured physical activity and sedentary time and all cause mortality: systematic review and harmonised meta-analysis. BMJ, 2019;366. 10.1136/bmj.l4570 10.1136/bmj.l4570 PMC6699591 31434697

[73] You Q, Yu L, Li G, He H, Lv Y (2022). Effects of different intensities and durations of aerobic exercise on vascular endothelial function in middle-aged and elderly people: a meta-analysis. Front Physiol.

[74] Bouchard C and Rankinen T, Individual differences in response to regular physical activity. 2001; ACSM symposium. 10.1097/00005768-200106001-00013 11427769

[75] Herbert P, Hayes LD, Sculthorpe NF, Grace FM (2017). HIIT produces increases in muscle power and free testosterone in male masters athletes. Endocr Connect.

[76] Sculthorpe NF, Herbert P, Grace F (2017). One session of high-intensity interval training (HIIT) every 5 days, improves muscle power but not static balance in lifelong sedentary ageing men A randomized controlled trial. Med. (United States).

[77] Bassey EJ, Fiatarone MA, O’Neill EF, Kelly M, Evans WJ, Lipsitz LA (1992). Leg extensor power and functional performance in very old men and women. Clin Sci.

[78] Earles DR, Judge JO, Gunnarsson OT (2001). Velocity training induces power-specific adaptations in highly functioning older adults. Arch Phys Med Rehabil.

[79] Hvid LG, Strotmeyer ES, Skjødt M, Magnussen LV, Andersen M, Caserotti P (2016). Voluntary muscle activation improves with power training and is associated with changes in gait speed in mobility-limited older adults - a randomized controlled trial. Exp Gerontol.

[80] Walker S, Haff GG, Häkkinen K, Newton RU (2017). Moderate-load muscular endurance strength training did not improve peak power or functional capacity in older men and women. Front Physiol.

[81] Rikli RE, Jones CJ (1999). Functional fitness normative scores for community-residing older adults, ages 60–94. J Aging Phys Act.

